# Multispectral Imaging Flow Cytometry with Spatially and Spectrally Resolving Snapshot-Mosaic Cameras for the Characterization and Classification of Bioparticles

**DOI:** 10.3390/mi13020238

**Published:** 2022-01-31

**Authors:** Paul-Gerald Dittrich, Daniel Kraus, Enrico Ehrhardt, Thomas Henkel, Gunther Notni

**Affiliations:** 1 Department of Mechanical Engineering, Group for Quality Assurance and Industrial Image Processing, Technische Universität Ilmenau, Gustav-Kirchhoff-Platz 2, 98693 Ilmenau, Germany; gunther.notni@tu-ilmenau.de; 2 Department of Nanobiophotonics, Leibniz Institute of Photonic Technology, Albert-Einstein-Straße 9, 07745 Jena, Germany; daniel.kraus@leibniz-ipht.de (D.K.); thomas.henkel@leibniz-ipht.de (T.H.); 3 Gesellschaft zur Förderung von Medizin-, Bio- und Umwelttechnologien e. V., Erich-Neuß-Weg 5, 06120 Halle (Saale), Germany; ehrhardt@gmbu.de

**Keywords:** multispectral, imaging flow cytometry, filter-on-chip, snapshot-mosaic, spectral imaging, population analysis, cultivation optimization

## Abstract

In the development and optimization of biotechnological cultivation processes the continuous monitoring through the acquisition and interpretation of spectral and morphological properties of bioparticles are challenging. There is therefore a need for the parallel acquisition and interpretation of spatially and spectrally resolved measurements with which particles can be characterized and classified in-flow with high throughput. Therefore, in this paper we investigated the scientific and technological connectivity of standard imaging flow cytometry (IFC) with filter-on-chip based spatially and spectrally resolving snapshot-mosaic cameras for photonic sensing and control in a smart and innovative microfluidic device. For the investigations presented here we used the microalgae *Haematococcus pluvialis* (HP). These microalgae are used commercially to produce the antioxidant keto-carotenoid astaxanthin. Therefore, HP is relevant to practically demonstrate the usability of the developed system for Multispectral Imaging Flow Cytometry (MIFC) platform. The extension of standard IFC with snapshot-mosaic cameras and multivariate data processing is an innovative approach for the in-flow characterization and derived classification of bioparticles. Finally, the multispectral data acquisition and the therefore developed methodology is generalizable and enables further applications far beyond the here characterized population of HP cells.

## 1. Introduction

Imaging Flow Cytometry (IFC) is an often used and powerful tool for the characterization of particles based on their intrinsic properties in microfluidic flow. Since the 1970′s, IFC has been used for a variety of applications in medicine, diagnostics, biotechnology, and microbiology [1,2,3,4]. For example, abnormalities in a cell population can be detected and identified [5]. Multispectral Imaging Flow Cytometry (MIFC) combines the spectral with spatially resolved morphological properties of particles. The data recorded in this way contain information about the material as well as spatial composition of the particles. A key challenge for the implementation of such systems, besides the fluidic focusing of the particles, is the design of the optical system. Previous approaches use complex optical arrangements to decompose the light into its spectral components [6]. For example, laser light sources [7], dispersive elements [8], optical filters [9], and special detector arrays [10] have been used. Due to the number of components, these systems are complex, difficult to operate and take up a lot of space.

To visualize the different cell compartments and their spatial distribution within the particles, often several fluorescent dyes are additionally used in parallel [11,12]. These staining procedures are time-consuming and usually alter the sample. Basiji et al. uses a so-called snapshot-method with multiple laser light sources and special dispersive elements to decompose the emitting light of the particles in ten separate fluorescent channels on two separate image sensors [11]. Di Caprio et al. applies a spectral scanning method based on the usage of linear variable filters to characterize the spectral and morphological properties of particles in-flow [9]. Within these scanning approaches the acquired image information must be aligned before generating a so-called multispectral data cube. The particles must not rotate when passing through the microfluidic system, because otherwise the spectral information cannot be assigned to the spatial positions within the particles. The here reported MIFC approach use a spatially and spectrally resolving snapshot-mosaic camera therefore, the rotation of the particle in microfluidic channel is not an issue. Using the MIFC approach for photonic sensing all spectral sub-images are acquired in parallel and don’t have to be aligned. 

Microalgae are increasingly becoming focused on in scientific research due to their widely distributed nature and wide range of potential applications. Already, bioactive substances [13,14,15,16] and other valuable materials [17,18] are produced in microalgae on an industrial scale. For the evaluation of the developed MIFC system we used the microalgae *Haematococcus pluvialis* (HP). HP is one of the microalgae species that is being used commercially to produce biotechnologically the antioxidant keto-carotenoid astaxanthin (Ax) [19,20]. 

State of the art for the development and optimization of such biotechnological cultivation processes is the morphological classification based on subjective microscopic evaluation and spectrometric point measurements. Spectrometric point measurements record a sum signal over numerous cells and does not contain information on population composition at the single cell level. Spectral and morphological properties of the same cells are not available at any time during these measurements. By using suitable optical measurement technology, the subjective assessment can be replaced by an objective measured value and thus a more precise indication of the prevailing conditions of the cells can be obtained. Furthermore, a much larger and thus more representative sample of particles can be analyzed and evaluated in a shorter time. In addition to process monitoring, the optimal harvesting time can thus be determined, which saves time, resources, and costs. 

Aim of this work is to open and practically evaluate the potential and suitability of filter-on-chip based spatially and spectrally resolving snapshot-mosaic cameras for the in-line characterization and classification of bioparticles in microfluidic flow.

## 2. Materials & Methods

### 2.1. Buffer and Sample Preparation

For the MIFC experiments, HP strain SAG34-1b (Culture Collection of Algae, Georg August University Göttingen, Göttingen, Germany) have been used. HP cells were prepared by cultivating HP strain SAG34-1b in standard culture medium BG1 [21]. The cells were previously cultivated in 500 mL glass vessels under the following cultivation parameters described in Table 1. The cultivation was performed for 10 days with continuous fumigation (gas mixture (1% CO_2_ (*v*/*v*)) under 350 mL/min flow volume. Before the MIFC measurements the cells were dissolved (1:1) experiment in 25% (w/vol) Ficoll^®^ 400 from Merck KGaA (Darmstadt, Germany). This prevents sedimentation of the cells during the experiments.

### 2.2. Microfluidic Chip Design and Fabrication 

The microfluidic chip devices consist of a two-layer system prepared by photolithography and wet etching of the glass wafers (thickness 0.7 mm) with hydrofluoric acid at a uniform etch depth of 100 µm. Nickel-chromium metallization was used as the mask. The micro-structured wafers were bonded face-to-face to create the functional geometries and closed microfluidic channels. The chip dimensions are 16.0 × 12.5 mm. The four fluid connection ports (diameter 520 µm) are fabricated by using ultrasonic drilling. The detection channel for flow-through image acquisition of the chip has a total width of 430 µm. The full channel height is 100 µm with a planar channel bottom.

### 2.3. Microfluidic Particle Focusing 

**Figure 1:**
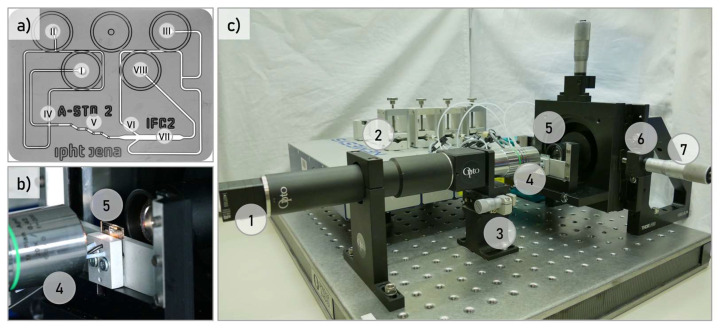
(**a**) Microfluidic chip with (I) Sample inlet, (II) Sheath fluid 1, (III) Sheath fluid 2, (IV) Flow Focusing Unit 1, (V) Flow rotation unit, (VI) Flow focusing unit 2, (VII) MIFC detection channel and (VIII) Waste outlet; (**b**) Chip integration; (**c**) Optical system with (1) Snapshot-mosaic camera, (2) Pressure-pump fluid management system, (3) Z-focus, (4) 20× Objective, (5) Microfluidic chip, (6) XY-linear stage, (7) LED white light source.

Basic requirement for image-based classification of particles within an image is that all particles are recorded with a high optical quality for the spatial and spectral value determination. Therefore, the particles are positioned in the focal plane of the optical system when passing the field of view (FOV) of the camera. For the experiments, a microfluidic chip with a fluid rotation unit designed for this purpose was used to focus all particles on the same horizontal plane (Figure 1a) [22]. Kleiber 2020 describes in detail the rotation and self-alignment of the particles in this microfluidic chip [23]. In the first step, the particles are focused on the center of the channel to form a vertical lamella. This lamella is rotated stepwise by 90° in the multi-layer rotation unit. This ends up in a horizontal lamella where all particles are in the same z-plane. Subsequently, the particles are focused again on the center of the channel to ensure that all particles pass the FOV of the snapshot-mosaic camera. The described microfluidic system works under laminar flow conditions with Reynolds numbers < 10 [24].

### 2.4. Optical Setup 

The optical setup consisted of a self-build microscope mounted on an optical bench (Figure 1c). The microscope was operated in transmitted light mode with a Köhler arrangement. An LED module 36 W 3500 K LED from CREE Inc. (Durham, NC, USA) was used. The microfluidic chip was integrated into an aluminum frame with the dimensions of a standard microscope slide (Figure 1b). This cartridge was integrated into an XY-stage from Märzhäuser Wetzlar GmbH & Co. KG (Wetzlar, Germany). A microscopy objective from Mitutoyo Corporation (Kawasaki, Japan) with a 20× magnification, numerical aperture (NA) of 0.42, was used. A filter-on-chip based spatially and spectrally resolving snapshot-mosaic camera MQ022HG-IM-SM4 × 4-VIS with 16 spectral channels from XIMEA GmbH (Münster, Germany) was used to record the data. The fluid management is controlled by three syringe pump modules from CETONI GmbH (Korbußen, Germany) and the associated neMESYS software. The chip was connected via two PTFE-HPLC tubing with an inner diameter (ID) 0.5 mm to two glass syringes (2500 µL) from ILS Innovative Laborsysteme GmbH (Stützerbach, Germany). These supply the flow focusing units 1 and 2 continuously with buffer. Additionally, tubing is connected to the outlet. 

The sample channel is connected to a PEEK-HPLC tubing ID 0.1 mm, which prevents cell aggregation in the sample channel. Each MIFC measurement requires approximately a 50 µL sample.

### 2.5. Camera Characterization Setup and Methodology

The setup which has been used for an extended characterization of the applied snapshot-mosaic camera works in accordance with the EMVA Standard 1288—Release 4.0 Linear from 16 June 2021 [25,26]. A Czerny-Turner Monochromator MSH-300 from Quantum Design GmbH (Darmstadt, Germany) has been used to illuminate the sensor surface for characterization. An Ulbricht-Sphere with a ODP97 coating from Gigahertz Optik GmbH (Türkenfeld, Germany) has been used to realize a reproducible and homogeneous arrangement in accordance with the requirements from the EMVA Standard 1288. Furthermore, a calibrated detector head RW-3705 for the measurement of irradiance in W/m^2^ also from Gigahertz Optik GmbH have been used [27]. The methods to measure and interpret the characterization results are described in [28,29,30].

### 2.6. Spatially and Spectrally Resolving Snapshot-Mosaic Camera 

As one of the key elements of the here presented approach is a filter-on-chip based spatially and spectrally resolving snapshot-mosaic camera from XIMEA GmbH (Münster, Germany). This camera uses Fabry-Pérot interference filters which have been directly applied within the CMOS-sensor manufacturing process. The used sensor within the camera is a CMV2000 CMOS-sensor from ams AG (Premstaetten, Austria) which have been equipped with the multispectral resolving interference filters by IMEC (Loewe, Belgium). The used camera has 16 spectral channels which lie between a usable wavelength range from 450 to 650 nm. First order maxima of the interpretable spectral channels are not arranged equidistantly. Due to the snapshot-mosaic arrangement of the spectral filters directly on the camera sensor surface, the 16 spectral channels are recorded simultaneously with one snapshot. The pixels are arranged periodically as a 4 × 4 matrix over the entire CMOS-sensor surface. Each pixel is covered with a filter and is thus assigned to a specific wavelength. All pixels of one wavelength are then combined to form a spectral sub image. 

### 2.7. Software Environment for Multivariate Data Processing

For the multivariate data processing and spectral model building we use the software fluxTrainer from LuxFlux GmbH (Reutlingen, Germany). This software allows complex multivariate data analysis and processing with spectral model building. With this software we developed a spectral model for the classification of HP cells on real multispectral data and perform data interpretation and result writing as complex .h5 files. To acquire and process data from snapshot-mosaic cameras we use .xml files with first- and second order filter wavelengths and the corresponding full width at half maximum (FWHM) values to handle the data the snapshot-mosaic camera is providing. 

## 3. Results & Discussion 

### 3.1. Data Acquisition and Raw Data Correction

**Figure 2:**
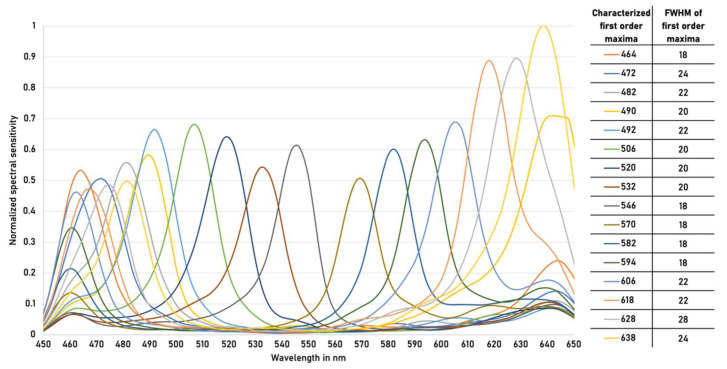
Characterized spectral sensitivity curves and their properties of snapshot-mosaic camera.

Prior to the experiments, an extended camera characterization was performed in accordance with [28,29,30]. This is necessary because the behavior of the used snapshot-mosaic camera concerning the spectral sensitivity differs from the generalized provided manufacturers data. We have updated the provided snapshot-mosaic camera specific .xml calibration file with the measured spectral sensitivity curves and the therefore derived first order maxima and their related FWHM values see Figure 2.

After connecting the snapshot-mosaic camera with the software using the updated .xml calibration file bright and dark reference images must be acquired as a first step before the actual processing. For setting up the multivariate data processing and spectral model building, different cell samples have been acquired, and the spectral model have been trained. The measurement image acquisition is done as a transmission image. This is a common standard in UV-VIS spectroscopy. For correct referencing, a bright and dark adjustment is performed to interpret the measurement image as a transmission image shown in Equation (1).

(1) $$T=\frac{I_{Sample}-I_{Dark}}{I_{Blank}-I_{Dark}}$$

where $I_{Sample}$ represents the captured image during the experiment, $I_{Dark}$ is the dark reference with the light source turned off, and $I_{Blank}$ is the bright reference in the microfluidic channel filled with buffer and without particles. 

**Figure 3:**
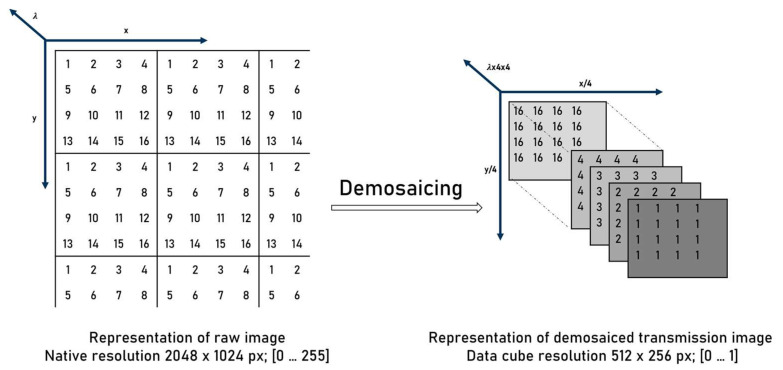
Resolution and value range of snapshot-mosaic camera images.

After transmission image calculation demosaicing is performed by the software environment shown in Figure 3.

The result are 16 spectral sub images, each with a size of 512 × 256 pixels. The resulting spatial resolution is thus limited. The acquisition with a snapshot-mosaic camera has the advantage that all spectral channels are recorded simultaneously. This means that the object only must be in the FOV of the camera at one point in time. A possible rotation of the objects while passing the FOV has no influence on the spectral evaluation compared to spectral scanning methods. Here, the spatial orientation of the object must not change when passing the FOV. 

The objects are recorded in transmission. Intensities are thus measured, i.e., photons per pixel. A correct substance quantity, i.e., a quantitative statement, can therefore only be made approximately. Small image features such as the phase objects or inner cell structures can thus only be resolved to a limited extent or not at all. For the training of the different spectral components, the inner cell structures are assigned to the different material classes. Due to the low spatial resolution, the phase objects are not considered in the calculation of the spectral components. Phase objects do not have any spectral information, but they are a part of the total cell. However, the measured spectral distribution within the cell allows an estimation of the percentage material composition of the cell. 

### 3.2. Multivariate Data Processing Model Building

A prerequisite for the reliable determination of the different cell stages is a robust and reproducible multivariate data processing and spectral model building for the automated in-line classification of HP cells. The spectral model is built by using reference spectra of HP cells from cell stages 1 and 2. HP cells mainly form two components chlorophyll (Chl) and Ax during the cultivation process. The spectral differentiation of the cell stages is based on the absorption maxima of Chl and Ax and their spatial and percentage distribution within the cells. Chl has two absorption maxima in the range of 400–500 and 630–690 nm. Ax has its absorption maxima in the range of 450–550 nm. The absorption bands of the two dyes partially overlap. However, in the range of 500–550 nm only Ax and in the range of 620–690 nm only chlorophyll can be detected [31,32]. The applied snapshot-mosaic camera is spectrally sensitive from 450 to 650 nm. Therefore, the ranges 500–550 nm and 620–650 nm are used for spectral discrimination between the cell stages. 

**Figure 4:**
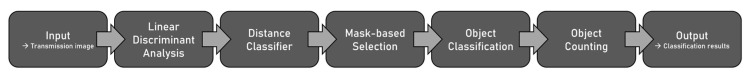
General structure of multivariate data processing and spectral model.

The multivariate data processing and spectral model follows a modular approach which means that different modules are logically linked (Figure 4). The definition of the spectral classes is done by supervised learning. 

For this purpose, definitions of the spectral signatures of the pure components are made. For the determination of the different cell stages, four spectral classes (red, green, background and cell wall) have been defined. For Chl, green cells from early stage 1 have been used. For Ax, cells from late stage 2. The spectral class of the image background is defined as free space without objects. The spectral characteristic of the cell wall corresponds to an empty cell envelope without dyes. These specifications ensure that the training data is based on a valid, robust, and reproducible data basis. 

The following section describes the spectral modeling procedure. After the definition of the pure components, linear discriminant analysis (LDA) is performed to reduce the spectral dimensions. By LDA each observation is assigned a score value. From this score, the group membership of each observation and the boundaries between the groups are calculated. If the spectral group membership of the observations is known, the feature variables are combined into a single discriminant variable shown in Equation (2).

(2) $$D_{LDA}=\beta_{0}+\beta_{1}X_{1}+\beta_{2}X_{2}+\cdots +\beta_{p}X_{p}$$

with $D_{LDA}$ discriminant variable, $\beta_{p}$ discriminant coefficients and $X_{p}$ feature variables. 

A Mahalanobis distance classifier was used to determine the spectral similarity or spectral distances shown in Equation (3). Due to the elliptical distribution of the data points. This classifier is used to group unknown image data into classes that have a minimum distance in multi-feature space. This makes the Mahalanobis distance particularly suitable for cluster analyses and is therefore used here.

(3) $$D_{MD}^{2}={\left(x-m\right)}^{T}\cdot c^{-1}\cdot \left(x-m\right)$$

$D_{MD}^{2}$ is the square of the Mahalanobis distance, x is the vector of the observation, m is the vector of mean values of independent variables and $c^{-1}$ is the inverse covariance matrix of independent variables.

**Figure 5:**
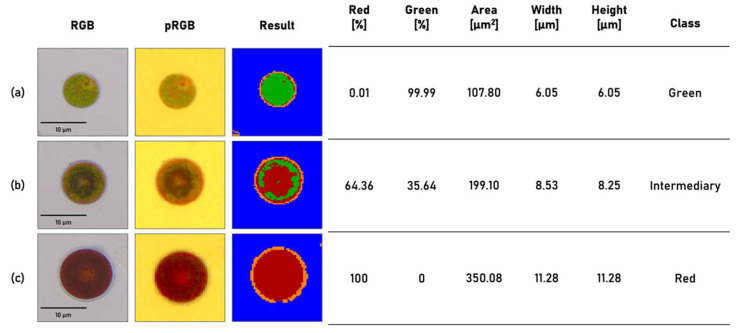
Results of multispectral data processing and spectral modeling. Microscope RGB image from reference camera (left). Processed pseudo RGB image from snapshot-mosaic camera (middle) Result image after multispectral classification (**a**) Green reference cell (**b**) Intermediary cell (**c**) Red reference cell.

After the spectral classes are assigned, mask-based selection is performed based on the classified individual pixels. Each pixel in the image is assigned to one of the defined classes based on the highest spectral similarity (Figure 5). Due to the filter arrangement, the snapshot-mosaic camera has less pixels per wavelength for the spatial scanning of the cell resulting in a lower spatial resolution compared to rgb-cameras. Due to the lower spatial resolution, only cell structures with a certain size can be reliably resolved. However, this is sufficient for the determination of the cell stages. The detected cell wall is shown in the false color image orange the detected background blue (Figure 5). For the further process of the detected cells only the Chl (green) and Ax (red) classified pixels are considered.

In addition to spectral properties, morphological properties are also included for classification. Only cells that correspond to the pre-defined parameters are recognized and classified. To exclude cell fragments as well as cell clusters, min and max cell sizes as well as cell areas are defined. All cells outside these boundaries are automatically excluded from the further processing. 

In the final step, all classified pixels within the object are counted and a percentage distribution of Chl to Ax is calculated. A cell is classified as green if more than 90% of the detected pixels were assigned to Chl. As a red cell, if more than 80% of the total area of the detected object was classified as Ax. If neither condition is met, the cell is classified as intermediate (late stage 1) (Figure 5). 

### 3.3. Single Cell Characterization in Flow

After sensor characterization and development of the spectral model, the MIFC system was practically evaluated for suitability to determine different HP cell stages in microfluidic flow. In addition to the reference experiments (C1–C4), mixtures (C5–C8) with unknown cell stage composition were examined to demonstrate that it is feasible to accurately analyze cell populations using the MIFC system. For statistical confidence, several thousand cells per sample have been analyzed (Table 2). The microfluidic flow rate was synchronized with frame rate of the snapshot-mosaic camera such that cells are captured only once as they pass through the FOV. This ensures that the cells are not counted multiple times during analysis. 

**Figure 6:**
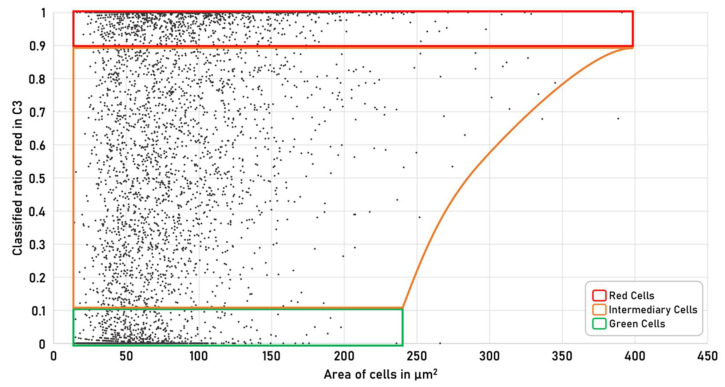
Scatterplot of classified spectral information and their morphological properties from the C3 heterogeneously distributed cell population. The red classified fraction within cells is plotted against cell size. The green marked area of dots represents green classified cells which contain only a small amount of Ax. The orange marked area of dots represents the intermediary classified cells which contain both Chl and Ax. The red marked area of dots represents the red classified cells which contain mainly Ax within the cells.

HP cells are cultivated in a two-stage cultivation process over 10 days. The details of cultivation conditions can be found in the “Material & methods” section. At the beginning of cultivation, the HP cells are in stage 1. In this stage, cells are green, motile, proliferate and therefore defined as green reference. Green cells have a smaller diameter compared to cells in the red stage. The difference in cell size as a function of the amount of Ax within the cell is shown in Figure 6. In the red stage, lipid vesicles are formed in which Ax is accumulated. This increases the size of the HP cells. As already described in Section 3.2, the cells are grouped into one of the three classes based on their predefined spectral and morphological properties. 

**Figure 7:**
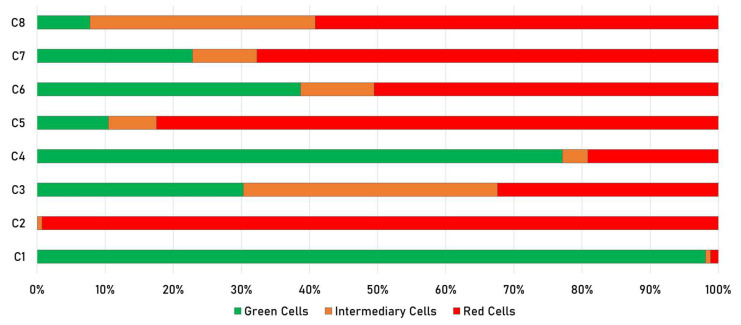
Results of MIFC measurements. (**C1**) Green reference control—cultivation under moderate illumination and nitrate in the culture. (**C2**) Red reference control—cultivation under light stress and nitrate deficiency. (**C3**) Cultivation under light stress. (**C4**) Cultivation under nitrate deficiency. (**C5**–**C8**) Unknown mixtures with cells from different cell stages.

Sample C1 was cultivated over the complied time under optimal conditions (moderate illumination and nitrate in the media) for the entire cultivation process. In the C1 Sample the cells mainly contain the green dye Chl (Figure 7C1). C2 represents the red reference. The cells are cultivated with the start of the experiment with light stress and nitrate starvation. The cells quickly enter cultivation stage 2, produce Ax and form round cysts (Figure 7C2). This is also confirmed by the increase in cell size compared to C1 (Table 2). During the transition from stage 1 to stage 2, the cells start to form Ax in their lipid vesicles and the spectral characteristics of the cells changes. 

The varying influence of the applied stress factors is visible in samples C3 and C4. In both experiments, only one stress factor was applied for the entire cultivation process. It appears that light stress induces a higher Ax product formation rate than nitrate deficiency. In C3, there are significantly more cells in intermediate or stage 2 than in C4 (Figure 7C3,C4). This is an indication that light stress is more effective than nitrate deficiency. The highest product formation rate is achieved by applying both light stress factors at the same time. The MIFC system was additionally evaluated with four unknown mixed samples (Figure 7C5–C8). These experimental approaches correspond to different time points during a cultivation process. By analyzing representative samples with serval thousand cells per sample, MIFC system can help to accurately monitor the cultivation process. A figure (Figure S1) and video sequence (Video S1) of the in-line classification can be found in the supplementary information. The experimentally determined MIFC results have been evaluated and confirmed by classical bright field microscopy. The reported MIFC system can thus be used as a supporting tool for in-line classification of cells in microfluidic flow.

## 4. Conclusions

In this reported work we present a novel realization for the characterization and classification of bioparticles in-flow using filter-on-chip based spatially and spectrally resolving filter-on-chip snapshot-mosaic cameras within the visible wavelength range. In this work we apply necessary sensor characterization for as close-to-reality as possible spatial and spectral value determination. The developed system can handle particles with sizes of 10 to 60 µm and is capable to classify up to 3600 HP cells per minute. Adaptation to other types of particles such as pollen, cells or microplastics would be feasible. The prerequisite for this is that the particles are in the size range of the system. This requires adaptations to the microfluidic chip and the corresponding multivariate data processing and spectral model building. Adaptions to the image acquisition system concerning camera and depending on the spectral properties of the investigated particles necessary dispersive elements or filters are not necessary because the snapshot-mosaic camera within its usable wavelength range is therefore already equipped and more flexible. The limiting factor of the MIFC system for the characterization of HP cells is neither hardware nor software, but the sample itself. The particle throughput depends strongly on the type of sample. HP cells tend to cluster at high particle concentrations. As a result, the described flow rotation unit (FRU) does not work correctly anymore. The cells must pass through the FRU individually to operate correctly. For particles that do not tend to cluster, the concentration can still be increased significantly. This also increases the particle throughput of the system. Among other things, it should be particularly emphasized that by using this type of snapshot-mosaic camera, the optical system can be simplified, and the size of the overall measurement system can be massively reduced. The multispectral data acquisition and the therefore developed methodology is generalizable and enables further applications far beyond the here characterized population of HP cells. With the MIFC system the spectral as well as the morphological features like the size of the cells can be determined simultaneously. Cells at the single cell level as well as whole cell populations can be spectrally and morphologically analyzed and counted. The multivariate data processing with spectral model building enables an in-flow characterization and classification of unknown mixtures of bioparticles. Furthermore, we evaluate the MIFC system successfully with 8 different sets of bioparticles. 

The reported MIFC system can thus be used as a supporting tool for in-line classification of cells in microfluidic flow. The MIFC system can be extended by a subsequent sorting unit [31,33]. This would enable the separation of targeted particles that exhibit specific characteristics for further investigation from a mixed population. Furthermore, the throughput and accuracy of the system could be improved by extending modern methods such as deep learning [34,35] or neural networks [36,37]. Through intelligent and efficient data reduction and subsequent processing, it is possible to evaluate these huge amounts of data in a short time even on standard computers.

## Figures and Tables

**Table 1:** Summary of the cultivation parameter.

	Culture Media		Illumination	
Nomenclature	1× BG-11 (+NaNO3)	1× BG-11 (−NaNO3)	Low Light (LL) ^1^ 16 h Daily	High Light (HL) ^2^ 24 h Daily
+N LL+C	x		x	
+N HL+C	x			x
−N LL+C		x	x	
−N HL+C		x		x

^1^ PFD, Spectral composition: 100 µE/m^2^s, 2700 K; 71 µE/m^2^s, 660 nm. ^2^ PFD, Spectral composition: 800 µE/m^2^s, 2700 K; 1065 µE/m^2^s; 450 nm; 724 µE/m^2^s, 470 nm.

**Table 2:** Conclusions of realized MIFC measurements.

ID	Label	Total Cells	Red Detected	Green Detected	Intermediary Detected	Ø Red Amount	Ø Green Amount	Ø Area in [µm^2^]	Red Classified	Green Classified	Intermediary Classified
C1	+LL2	8326	96	8170	60	0.015	0.985	54.75	1.2%	98.1%	0.7%
C2	−HL2	9553	9483	6	64	0.997	0.003	85.03	99.3%	0.1%	0.7%
C3	+HL2	5549	1798	1680	2071	0.565	0.435	77.00	32.4%	30.3%	37.3%
C4	−LL2	1665	319	1284	62	0.211	0.789	57.39	19.2%	77.1%	3.7%
C5	Mix1	2919	2407	306	206	0.864	0.136	71.52	82.5%	10.5%	7.1%
C6	Mix2	2691	1360	1041	290	0.567	0.433	73.28	50.5%	38.7%	10.8%
C7	Mix3	1999	1354	456	189	0.733	0.267	79.75	67.7%	22.8%	9.5%
C8	Mix4	2189	1295	170	724	0.776	0.224	86.44	59.2%	7.8%	33.1%

## Data Availability

Not applicable.

## Supplementary Materials

The following supporting information can be downloaded at: https://www.mdpi.com/article/10.3390/mi13020238/s1. Figure S1: Result images of snapshot-mosaic camera—in-line classification and rgb-camera—reference. Video S1: In-line HP cell classification.
