# Supplementary figures and images for: Multispectral Imaging Flow Cytometry with Spatially and Spectrally Resolving Snapshot-Mosaic Cameras for the Characterization and Classification of Bioparticles
Source: Micromachines (Basel). 2022 Jan 31;13(2):238. doi: 10.3390/mi13020238 (PMC8879709; doi:10.3390/mi13020238)

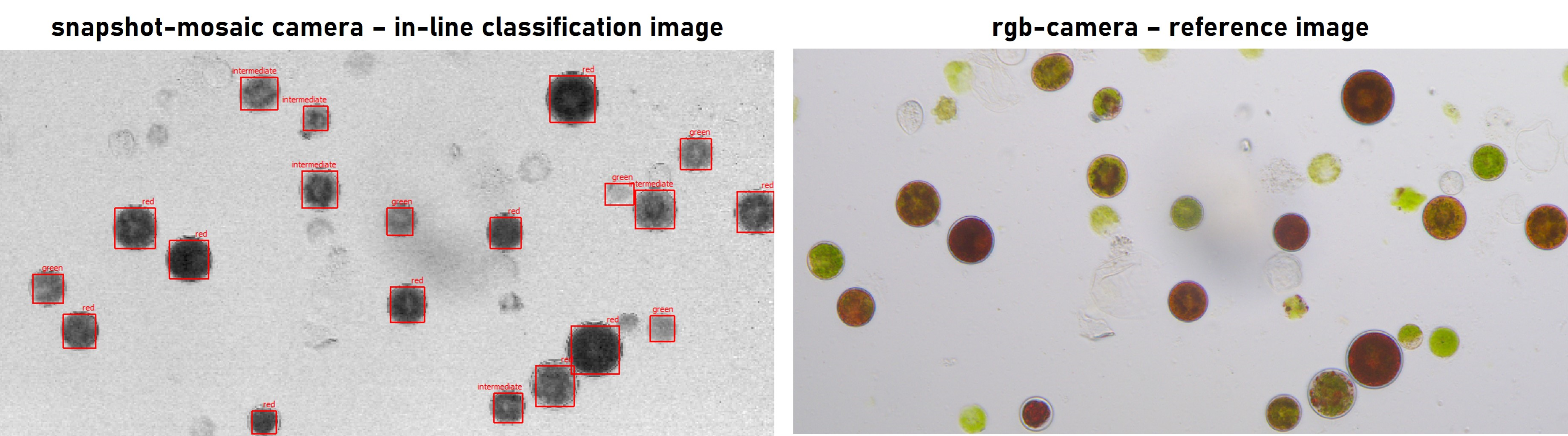

Supplement: Supplementary file 1 [file micromachines-13-00238-s001.zip › Figure S1.tif]
